# The Impact of Breakthrough Innovations on the Export Performance of SMEs in Developing Countries: The Moderating Role of Institutional Factors

**DOI:** 10.3389/fpsyg.2022.888697

**Published:** 2022-05-12

**Authors:** Hammad Bin Azam Hashmi, Cosmina L. Voinea, Ward Ooms, Marjolein C. J. Caniëls

**Affiliations:** Faculty of Management, Open Universiteit, Heerlen, Netherlands

**Keywords:** breakthrough innovations, technology-based innovations, market-based innovations, export performance, SMEs, institutional environment specificity and enforceability

## Abstract

Although few studies examine the implications of breakthrough innovations in the exporting context, we have little understanding about how contingent factors in the developing countries shape the breakthrough innovations–export performance link. Thus, this study aims at examining the impact of breakthrough innovations (i.e., technology-based innovations and market-based innovations) on the export performance of SMEs in developing countries, while studying the role of contingent factors, i.e., institutional environment specificity and enforceability. The data were collected from a sample of 410 SMEs in Pakistan. Hypotheses were tested through structural equation modeling in AMOS 20. The results reveal that both technology-based and market-based innovations have a positive impact on the export performance of SMEs in terms of strategic export performance and economic export performance. Second, institutional environment specificity and enforceability enhance the impact of breakthrough innovations on the export performance of SMEs.

## Introduction

Studies confirm that innovation strategies and technology enable firms to expand and compete internationally (Amankwah-Amoah et al., 2018; Bortoluzzi et al., 2018; Pino Soto, 2018; Donbesuur et al., 2020; Vuorio et al., 2020). For instance, researchers have argued that formulating new technological strategies and adopting present ones are critical for firms’ productivity and the knowledge economy (Wamboye et al., 2015; Wong, 2015; Schniederjans, 2017). Particularly, scholars are giving much intention to the influence of innovation on export performance (Azar and Ciabuschi, 2017; Silva et al., 2017; Tavassoli, 2018; Wadho and Chaudhry, 2018; Reçica et al., 2019; Edeh et al., 2020; Enjolras et al., 2020; Wu et al., 2021). However, few have examined the link between breakthrough innovations and export performance (e.g., Silva et al., 2017). Breakthrough innovations refer to groundbreaking products and services which promise customers’ extraordinary performance and help firms achieve massive cost reduction (Wang and Feng, 2020). Breakthrough innovations are different from the incremental innovations (i.e., a minor improvement in product and technology to enhance current performance) in a way that they introduce new technologies, offer considerably greater benefits to the customers in comparison with existing products, and substantially change the current usage and consumption patterns (Zhou and Li, 2012; Cheng and Chen, 2013; Byun et al., 2021). Breakthrough innovations include technology-based innovations and market-based innovations (Zhou et al., 2005). Technology-based innovations refer to the technological advancements that include developing and implementing new and high-quality technical innovations replacing old and inferior alternatives (Zhou et al., 2005; Ulaga and Eggert, 2006). However, market-based innovations mean departing from existing market segments or mainstream markets and serving new markets.

Although we may find the role of breakthrough innovations in influencing export performance from the perspective of developed countries (Silva et al., 2017), the impact of breakthrough innovations on small- and medium-sized enterprises (SMEs) export performance in developing contexts is yet to explore. The rationale behind is that the ongoing globalization and changing customers’ needs have led SMEs in developing context to seek new ways and alter existing ones to survive and maintain a competitive position in local and international markets. Specifically, as compared to developed economies that have strong legal systems and efficient capital markets (Khanna and Palepu, 1999), developing countries face issues of weak legal regimes, corrupt political systems, administrative inefficiencies, and lack of transparency. Studies argue that such institutional factors can both hinder or promote firms’ export activities (Adomako et al., 2019; Donbesuur et al., 2020; Nuruzzaman et al., 2020; Hernández et al., 2021). Since SMEs are resource-constrained and thereby facing high innovation costs, they seek institutional support to protect their innovations and inventions. Given the divergent contingency of institutional framework in a developing country, it is important to explore the role of the institutional environment while examining the influence of breakthrough innovations on the export performance of SMEs in developing contexts. For this purpose, this study adopts two important dimensions of institutional environment, i.e., institutional environment specificity and enforceability (Ngo et al., 2016) as important contingent factors that may influence the relationship between breakthrough innovations and export performance of SMEs. These two dimensions together explain how the institutional environment can protect the SMEs’ innovations and property rights through provisions and implementation of rules and regulations (Ngo et al., 2016; Donbesuur et al., 2020). It means a greater level of institutional environment specificity and enforceability can help SMEs protect their innovations and consequently improve their export performance. Hence, we posit two questions: first, what is the role of breakthrough innovations in improving the export performance of SMEs in developing countries? Second, how institutional environment specificity and enforceability influence the breakthrough innovations–export performance link? Therefore, we aim to examine the impact of breakthrough innovations on export performance of SMEs with moderating role of institutional environment.

The current study adds value to the export business literature and innovation literature, in the following three major ways. First, drawing on the institutional theory and institutional literature framework (Bruton et al., 2010; Hessels and Terjesen, 2010; Ngo et al., 2016; Amankwah-Amoah et al., 2018; Donbesuur et al., 2020), we extend the institutional environment by examining the moderating influence of institutional environment specificity and enforceability on the relationship between breakthrough innovations and export performance. In this regard, we offer a comprehensive understanding by examining the moderating influence of the institutional environment separately for both technology-based and market-based innovations in relation to the export performance of SMEs. Second, the current study extends the notion of dynamic capabilities to export business literature by highlighting the influence of SMEs’ breakthrough innovations in terms of technology-based and market-based innovations on export performance in developing contexts. Finally, we tested our proposed model in the unique developing setting of Pakistan. This is an important contribution as little focus has been given to understand how SMEs in developing context enhance their export performance through their breakthrough innovations. Also, to understand how the institutional environment of a developing country can influence the innovation–export performance link.

After the introduction and research background, the structure of the paper is as follows: First, the literature review and conceptual model of the study are provided. Second, the methodology is elaborated, explaining sampling design and measurement of the constructs. Third, the findings of the current study are provided. That is followed by a discussion of the findings and conclusion.

## Theoretical Background

### Dynamic Capability-Based View and Breakthrough Innovations

Previous research emphasizes the importance of dynamic capabilities for the firms in achieving a competitive advantage, particularly during changing environments (Teece and Pisano, 2003; Liao et al., 2009; Adeniran and Johnston, 2012; Mikalef and Pateli, 2017; Albort-Morant et al., 2018; Ferreira et al., 2020; Han and Zhang, 2021). Dynamic capabilities explain how firms acquire, alter, incorporate, and recombine resources to create firm value. Hence, these capabilities cover both managerial and organizational routines, which assist firms in the management of resources. The dynamic capabilities may help firms develop working capital that further influences company performance (Rus and Achim, 2020). The dynamic capability-based view has been employed previously to describe many organizational processes and outputs, including innovation capabilities (Schoemaker et al., 2018; Ferreira et al., 2020), cognitive processes (Easterby-Smith et al., 2009), and firms’ decision regarding internationalization (Arikan et al., 2019). Considering these tenets of dynamic capabilities and the extant research about the influence of innovation on internationalization (Lewandowska et al., 2016; Tavassoli, 2018; Reçica et al., 2019; Edeh et al., 2020; Enjolras et al., 2020; Wu et al., 2021), the current study conceptualizes breakthrough innovations as firms’ capabilities, enabling SMEs’ to enhance their export performance. Hence, we may assume that dynamic capability offers sound theoretical support to explain the influence of breakthrough innovation on international performance.

### Breakthrough Innovation and Export Performance

Generally, innovation involves the process of developing and executing an idea in the form of a product, process, or behavior (Damanpour and Evan, 1984; Damanpour, 1996). Innovation is a complex phenomenon and covers several activities, including managerial and administrative processes; product development; and organizational structures (Damanpour and Aravind, 2012). Whereas breakthrough innovations refer to the process by which required knowledge is created and associated with other current or new knowledge in marketable terms that has the ability to disrupt the existing market and/or develop new markets (Mooty and Kedia, 2014, p. 223). It is commonly argued that breakthrough innovations recombine diverse and distant knowledge bases (Kaplan and Vakili, 2015) and allow firms to deep dive into a particular business by exploring complex issues (Kamuriwo et al., 2017). Breakthrough innovations in the form of a novel and unique product may generate a new business domain, offering new benefits and attracting new markets (O’Connor, 2008). Some of the examples of breakthrough innovations include automatic welding machines, autopilot cars backed by artificial intelligence (in-progress), and microfinance loans. The above-mentioned characteristics suggest that breakthrough innovation is a complex phenomenon, and thereby it may require innovation capabilities and a new knowledge base.

Wang and Feng (2020) identified that there are two significant predictors of technological innovation breakthroughs, i.e., technological factors and market factors. Originally, Benner and Tushman (2003) categorized technological innovation breakthroughs into technology innovation and market innovation after comparing new technologies with existing technologies and evaluating new technologies in relation to existing market segments. Later on, Zhou et al. (2005) extended this conceptualization into two groups: technology-based innovations and market-based innovations. The former group is defined as those innovations that drive through new techniques, involving the most advance and cutting edge R&D functions; however, the latter type of breakthrough innovations involve offering simple solutions to the emerging market segments that may bring substantial improvements in the markets. In the same way, Govindarajan et al. (2011) contended that technology-based innovations follow new material techniques to be incorporated into products, whereas market-based innovations are typically designed for emerging markets or new customer segments with a focus on existing techniques. The classification of breakthrough innovations by Zhou et al. (2005) is well accepted in the current literature. For example, Silva et al. (2017) has recently reported a positive association between breakthrough innovations (i.e., technology-based and market-based innovations) and export performance in the Portuguese context. Similarly, the same classification was explored in relation to organization performance (Wang and Feng, 2020) and innovation performance (He et al., 2021). Consequently, the current study follows Zhou et al.’s typology of breakthrough innovations while examining the link between breakthrough innovations and export performance.

#### Technology-Based Innovations and Export Performance

The role of innovation in getting and maintaining competitive advantages is not new in the literature (Day and Wensley, 1988; Hunt and Morgan, 1995). Innovation is the process through which firms can effectively use assets and capabilities, and translate them into high-performance outcomes (Reed and Defillippi, 1990; Barney, 1991). Technological innovations in the context of SMEs can be referred to as any unique discovery that substantially extends current technologies and has a novel value as compared to existing products (Wang and Feng, 2020). Besides, technology-based innovations can be found as inputs, processes, and outcomes (Bagheri et al., 2019).

Technology-based innovations are key determinants of firm performance (Urbancova, 2013), as it has been argued that technological innovations like the introduction of new products and processes enable SMEs to successfully compete internationally (Filipescu et al., 2009; Bagheri et al., 2019; He et al., 2019). It is credited to the fact that advanced technological innovations influence firms’ decisions about internationalization that may enhance profitability and exports (Bagheri et al., 2019; Donbesuur et al., 2020; Rusa et al., 2021). SMEs that frequently introduce new products encompassing advanced technologies have a greater ability to improve processes associated with administration, management, and marketing (Adam et al., 2017; Afriyie et al., 2019; Expósito and Sanchis-Llopis, 2019). This ability may allow firms to perform well in the export industry. Recently, Radicic and Djalilov (2019) determined that SMEs which invest in technology-based innovations have better export performance. Besides, Love et al. (2016) reported a positive association between technological innovation and SMEs’ export performance.

*Hypothesis* 1: Technology-based innovations are positively associated with the export performance of SMEs in terms of economic export performance and strategic export performance.

#### Market-Based Innovations and Export Performance

Market-based innovations include covering new markets or market expansions with a focus on creating superior customer values (Xu et al., 2017). Due to the turbulent market environment, companies often try to search for new markets for their market-based innovations. In this regard, they exploit their interactive and learning abilities to get implicit and complex knowledge about the new markets at the local and international level to determine space for their market-based innovations (Tsai and Wang, 2017). Also, market-based innovations pave the better way to respond to the demands of low-end markets and emerging markets (Kim and Atuahene-Gima, 2010; Zhang and Qiu, 2013), as market-based innovations enable firms to gather advanced knowledge and technology during fierce competition in the markets and use this knowledge and technology to introduce new products and processes, hence allowing them to capture a good position in the emerging markets. According to Zhou et al. (2005), market-based innovations improve firm performance as these innovations offer the first-mover advantage and are well embraced by emerging markets. Generally, firms that have the potential to generate and examine the customers’ and competitors’ knowledge can exploit new and emerging markets through breakthrough innovations. And this may eventually lead to better export performance. Therefore, we can argue that market-based innovations also play an important role in enhancing the export. So, we can conclude that:

*Hypothesis 2*: Market-based innovations are positively associated with the export performance of SMEs in terms of economic export performance and strategic export performance.

### Institutional Factors

We have adopted institutional theory to better explain the influence of the institutional environment (institutional specificity and enforceability) on the relationship between breakthrough innovations and export performance. The characteristics of the institutional theory reflect that institutional frameworks and arrangements whether it is external or internal to the firm may impact business transaction mechanisms, strategic decisions, and subsequent performance outputs, including the chances of being involved in exporting and international activities (Bruton et al., 2010; Volchek et al., 2013). Studies have advanced the perspective that governance and institutional systems are key determinants of firms’ competitive performance, particularly important for the firms operating in developing and emerging economies (Adomako et al., 2019; Donbesuur et al., 2020). Consequently, a firm’s domestic and international success significantly depends on the institutional environment or institutional arrangement.

Considering the influence of breakthrough innovation on export performance (Silva et al., 2017) and taking the support from the international business literature (Zhou and Poppo, 2010; Ngo et al., 2016; Donbesuur et al., 2020), the current study has adopted institutional environment specificity and enforceability as significant boundary conditions. There are three important reasons behind adopting these boundary conditions. First, the presence and absence of a strong institutional environment are critical to a firm’s innovation activities (Barasa et al., 2017). It has been observed that SMEs in developing markets often face innovation costs (Safari and Saleh, 2020). Thus, legal systems like intellectual property rights’ specificity and enforceability that are supposed to safeguard the innovations will be the most suitable way to enhance export performance. Hence, the benefits coming through SMEs’ innovations are greatly influenced by the availability or absence of legal systems and the protection of innovations. Second, either a strong or weak institutional environment has a significant influence on firms’ exporting performance or internationalization activities (LiPuma et al., 2013; Adomako et al., 2019). So, institutional theory can allow us to explain that when breakthrough innovations are in a position that would enhance exporting performance. Third, the context of developing markets is itself an important rationale behind investigating the impact of the institutional framework on the relationship between breakthrough innovations and exporting performance. About developed economies, emerging and developing economies often face institutional voids that refer to the situation when “institutional arrangements that support markets are absent, weak, or fail to accomplish the role expected of them” (Mair and Marti, 2009). These voids are characterized by several institutional faults such as corruption, political instability, violation of intellectual property rights (Khanna and Palepu, 1999), all of which may influence a firm’s exporting strategies and performance.

#### The Moderating Role of Institutional Environment Specificity

The institutional environment specificity refers to the degree to which firms’ ownership rights of innovation and invention in the form of new products, processes, and administrative or organizational methods are explicitly stated and defined in the existing rules and regulations of a particular context (Griffith and Zhao, 2015; Ngo et al., 2016). We argue that institutional environment specificity increases the impact of breakthrough innovations on the export performance of SMEs. The rationale behind our argument is that when firms’ innovation practices are well protected by the formal laws and regulations, they may get an assurance from the institutions. Thus, firms become more confident about the laws and actively engage in breakthrough innovations. More specifically, firms have to bear high costs and investments associated with the innovative practices as these practices aim to disrupt existing ways to develop new products and introduce new processes (Maekelburger et al., 2012; Askenazy et al., 2013). So when the institutional specificity is high, these innovations would be safeguarded and protected, thereby allowing firms to get the maximum benefits from their innovation ventures (tech- and market-based). Consequently, the institutional environment specificity provides firms the peace of mind and latitude with which they can freely involve in export activities, improving export performance. Besides, high institutional environment specificity may enable firms to reduce transaction costs and manage uncertainty in foreign markets (Maekelburger et al., 2012; Ngo et al., 2016), which can further improve export performance. Accordingly, we state that:

*Hypothesis 3a*: Institutional environment specificity strengthens the impact of technology-based innovations on the export performance of SMEs.

*Hypothesis 3b*: Institutional environment specificity strengthens the impact of market-based innovation on the export performance of SMEs.

#### The Moderating Role of Institutional Environment Enforceability

The institutional environment enforceability can be defined as the degree to which firms’ ownership rights of innovation and invention in the form of new products, processes, and administrative or organizational methods are assured and effectively enforced by the relevant authority in the home country (Acemoglu and Johnson, 2005; Bai et al., 2016; Ngo et al., 2016). The lack of strong institutional enforceability in the home country reflects that country’s legal system is not able to enforce rules and regulations, which are supposed to protect firms’ ownership rights of innovation. Thus, we contend that besides stating and specifying the rules and regulations to protect innovations and firms, these rules must be enforced to build absolute confidence in institutions. Therefore, institutional enforceability can be an important environmental factor that influences the relationship between breakthrough innovations and export performance. Where institutional enforceability is guaranteed, it may enhance the level of firms’ trust in formal institutions and also limit unfair market practices (Peng, 2003; Geleilate et al., 2016). The credibility of the institutions is a significant driver of firms’ internationalization activities (Geleilate et al., 2016). Therefore, when firms get the signal of strong institutional enforceability, they consider the home environment safe and protected, and feasible to invest in innovative projects (Chadee and Roxas, 2013; Ngo et al., 2016; Urban, 2016), leading to improved export performance. Besides, the authors suggested that institutional environment enforceability may help firms reduce operating costs in foreign markets and safeguard intellectual property rights (Meyer and Nguyen, 2005), hence, we conclude that.

*Hypothesis 4a*: Institutional environment enforceability enhances the impact of technology-based innovations on the export performance of SMEs.

*Hypothesis 4b*: Institutional environment enforceability enhances the impact of market-based innovations on the export performance of SMEs.

**Figure 1:**
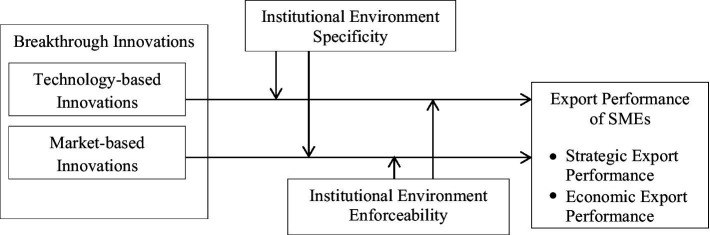
Proposed model.

The conceptual model is elaborated in Figure 1.

## Methodology

### Research Setting and Sample

To examine study hypotheses, we used a sample of SMEs operating in Pakistan. There are two important reasons behind considering Pakistan as the research setting. First, SMEs in Pakistan are greatly contributing to the economy by sharing 40% in GDP, creating 80% employment in the country, and managing 30% of export activities (Khalique et al., 2015; Ali Qalati et al., 2021). Hence, the export literature may get benefits from the research that examines how SMEs in developing economies continue to internationalize with their breakthrough innovations and what are the significant contributors to their performances in international markets. Second, in recent times, Pakistan has achieved promising improvements in maintaining a sound institutional environment to support and protect innovative practices of the firms (Trade Representative, 2016). The laws are specified to protect intellectual property rights (IPR) such as patents, trademarks, copyrights and to provide investors solid guarantees regarding the protection of intangible assets (Janjua et al., 2019). This is credited to the fact that Pakistan has made good efforts to improve IPR laws and enforcement by implementing key aspects of the “Intellectual Property Organization of Pakistan Act of 2012 (Trade Representative, 2016). These endeavors include establishing tribunals for IPR protection and a schedule to amend major IPR laws, creating public awareness about IPR laws, and the imminent implementation of the IP enforcement rules. With these constructive roles of SMEs in the economy and economic outlooks, the country offers a relevant context to examine study propositions. Consequently, the information coming from this context may contribute significantly to the export business literature.

Considering our study context and following previous studies (Silva et al., 2017; Gerschewski et al., 2018; Ali Qalati et al., 2021), the following criteria have been adopted to finalize an appropriate random sample from the sample frame. We focused on the export venture as a unit of analysis: (i) firms that offer single or multiple products to be exported and (ii) engage in innovative practices. The information about registered SMEs was gathered through listings available at Lahore and Karachi Stock Exchange, all Chamber of Commerce in Pakistan, Jamal Yellow pages, and the Punjab Directory of Industrial Establishment. The sample was randomly drawn from four major provinces of Pakistan, including Punjab, Sindh, Khyber Pakhtunkhwa, and Balochistan. Thus, we were able to select 514 firms as a final sample, which fulfilled our sampling criteria. This sample covered six major industries, including textile, sports, food, leather, furniture, and metal. An online survey was done through the distribution of 514 closed-ended questionnaires in a period of five months. We sent an online survey through an email to potential respondents with a cover letter, including a confidentiality clause and an explanation of the study purpose. The online survey can help gather information from a large population and thereby provide greater statistical power (Manfreda et al., 2008). Among distributed questionnaires, we received 423 responses, and from which 13 responses were excluded due to incomplete information. Hence, 410 responses were available for the final analysis. Table 1 provides information about the sample profile.

**Table 1:** Sample information.

Industry	Percentage (%)	Number of firms
*City/district*		
Lahore	19.51	80
Multan	4.39	18
Gujranwala	17.56	72
Karachi	28.05	115
Gujarat	3.41	14
Hyderabad	2.93	12
Sialkot	5.37	22
Sheikhupura	3.90	16
Faisalabad	14.88	61
*Industry category*		
Textile	21.95	90
Sports	15.85	65
Leather	14.63	60
Furniture	13.41	55
Food	14.15	58
Metal	9.27	38
Others	10.73	44
*Firm size*		
Small (10–50 employees)	44	180
Medium (51–250 employees)	56	230
*Export intensity*		
High (>50% export sales)	26.82	110
Moderate (25–49% export sales)	31.70	130
Low (<24% export sales)	41.46	170

### Non-response Bias

To handle non-response bias, we compared early responses (initially received 60% responses) and late responses (the remaining 40%) with all items measuring main study constructs, number of employees, and export intensity. We found no significant differences under this comparison, indicating that there is no significant issue of non-response bias in this study (Armstrong and Overton, 1977).

### Common Method Bias

To manage common method bias (CMB), this study maintained the conciseness and simplicity in the questionnaire. Moreover, we used reverse coded items with different dimensions spreading across the questionnaire. By doing this, we ensured consistency in the questionnaire. To further address the bias, we ran ex post statistical technique. For that purpose, we conducted partial correlation and Harman’s single factor test to check CMB (Podsakoff et al., 2003). The Harman’s test reveals that all items were loaded on single factor, showing variance of 19%. Hence, there is no issue of CMB as the total variance is less than 50%. The partial correlation test shows that addition of the marker variable did not influence the hypothesized paths as well as their significance. Lately, multicollinearity statistics reveal that there is no problem of multicollinearity as the variance inflation factor (VIF) is smaller than 3 (Johnson and Lebreton, 2004).

### Measures

We adopted measures from previous studies in the developing context of Pakistan. The detail about measures is given in Appendix A. The construct of export performance, including economic and strategic export performance, was adapted from Silva et al. (2017). They previously used scales from the study of Zou et al. (1998) and Morgan et al. (2004). The economic export performance was measured on a seven-point scale: 1 for “much worse” and 7 for “much better,” while the strategic export performance was measured on the seven-point scale: 1 for “strongly disagree” and 7 for “strongly agree.” The construct of breakthrough innovation in terms of technology-based innovations and market-based innovation was adapted from the study of Zhou et al. (2005). The anchor used to measure breakthrough innovation includes a seven-point scale: 1 for “strongly disagree” and 7 for “strongly agree.” The constructs of institutional environment specificity and enforceability were adapted from previous studies (Zhou and Poppo, 2010; Ngo et al., 2016; Donbesuur et al., 2020). For institutional environment specificity, the respondents were directed to indicate to what extent they perceive about the existence of institutional rules and regulations about their innovations on a seven-point scale (1 for “non-existing” and 7 for “prevalent”). Similarly, we asked respondents to indicate to what extent they perceive the level of enforcement regarding the protection of their innovations on a seven-point scale (1 for “strongly disagree” and 7 for “strongly agree”).

## Findings

### Descriptive Statistics

Descriptive statistics provide a level of respondents’ agreement regarding study constructs. Table 2 shows that respondents have a positive perception of technology-based innovations and market-based innovation with a mean value greater than 3.5. Near to respondents, technology-based innovations are more important as the mean value (mean = 4.63) is greater than the mean value (3.95) of market-based innovation. Moreover, respondents demonstrate a good level of agreement with export performance, institutional environment specificity, and enforceability with a mean value greater than 3.5.

**Table 2:** Respondents’ perceptions.

	N	Minimum	Maximum	Mean	SD
Export performance	410	1.00	7.00	4.5733	1.23475
Technology-based innovations	410	1.00	7.00	4.6390	1.29681
Market-based innovation	410	1.00	7.00	3.9488	1.51645
Institutional environment specificity	410	1.00	7.00	5.3040	1.18986
Institutional environment enforceability	410	1.00	7.00	4.8143	1.12408
Firm size	410	1.00	2.00	1.5610	0.49687

### Measurement Model

We employed two-stage structural equation modeling (SEM) through AMOS 21 to determine the construct validity and to examine hypothesized paths. During the first stage, confirmatory factor analysis (CFA) was conducted to assess the discriminant and convergent validity. In this regard, the measurement model was developed. The construct of export performance was considered as a second-order construct in the measurement model as it contains two dimensions, i.e., economic export performance and strategic export performance (Davcik, 2014). The model fitness indices show that the measurement model is fit with all fit indices fall under the recommend range, i.e., Chisq/df < 3 (1.96); CFI > 0.90 (0.98); NNFI > 0.90 (0.97); RMSEA < 0.08(0.049) (Bagozzi and Yi, 1988; Browne and Cudeck, 1992; Bollen and Hoyle, 2012; Hair et al., 2014). Bagozzi and Yi (1988) suggested that the convergent validity of the constructs will be satisfied if the value of standardized factor loadings >0.5, composite reliability (CR) > 0.7, and average variance extracted (AVE) > 0.5. Table 3 shows that convergent validity is satisfied as standardized factor loadings are greater than 0.5, CRs are greater than 0.7 and AVEs are greater than 0.5 for all constructs.

**Table 3:** Convergent validity.

Constructs	Dimension	Items	Factor loadings	Composite reliability (CR)	Average variance extracted (AVE)
Export performance	Economic export performance	EXP1	0.762	0.872	0.695
		EXP2	0.916		
		EXP3	0.815		
	Strategic export performance	SXP1	0.727	0.878	0.708
		SXP2	0.845		
		SXP3	0.939		
Technology-based innovations		Tech1	0.86	0.878	0.652
		Tech2	0.913		
		Tech3	0.875		
		Tech4	0.518		
Market-based innovation		Mar1	0.713	0.908	0.713
		Mar2	0.838		
		Mar3	0.917		
		Mar4	0.895		

The discriminant validity of the constructs was assessed by following the method of Fornell and Larcker (1981). They determined that discriminant will be satisfied if the square root of AVE of a particular construct is greater than the corresponding correlation among the variables. Table 4 reveals that the value of the square root of AVE on the diagonal is greater than the corresponding correlation among the variables. So, discriminant validity is also satisfied.

**Table 4:** Discriminant validity.

	Market-based innovation	Economic export performance	Strategic export performance	Technology-based innovations	Firm size
Market-based innovation	**0.844**				
Economic export performance	0.500	**0.833**			
Strategic export performance	0.144	0.130	**0.841**		
Technology-based innovations	0.370	0.543	0.180	**0.807**	
Firm size	0.013	0.020	0.038	0.037	**N/A**

*Square root of AVEs are presented in diagonal.*

### Structural Model and Hypotheses Testing

**Figure 2:**
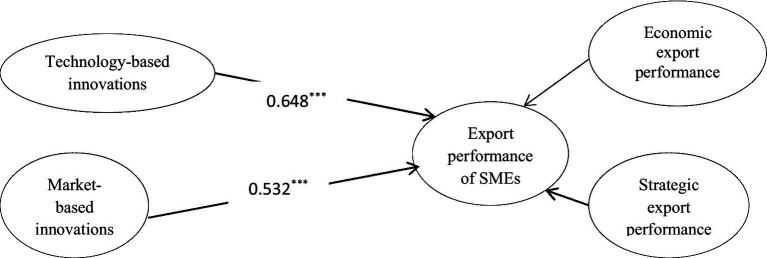
Standardized paths. ^*^p < 0.05; ^**^p < 0.01; ^***^p < 0.001.

After the assessment of the measurement model, we developed a structural model to examine the hypothesized paths. The model is fitted with all fit indices fall under the recommended range, i.e., Chisq/df < 3 (1.94); CFI > 0.90 (0.98); NNFI > 0.90 (0.97); RMSEA < 0.08(0.048) (Bagozzi and Yi, 1988; Browne and Cudeck, 1992; Bollen and Hoyle, 2012; Hair et al., 2014). Findings about the hypothesized paths are given in Table 5. H1 posits that technology-based innovations are positively associated with the export performance of SMEs in terms of economic export performance and strategic export performance. It is accepted as the β-estimate (0.648) from technology-based innovations to export performance is significant with *p* < 0.05 (Table 5). This finding is in agreement with previous studies (Zhou et al., 2005; Paladino, 2007; Silva et al., 2017; Wadho and Chaudhry, 2018). Technology-based innovations enhance customer value by offering advanced technologies, replacing inferior alternatives. Thus, it may lead to a better economic and strategic position internationally. Besides, uniqueness and newness among products and services may improve firms’ ability to satisfy customers’ latent needs and thereby provide a competitive advantage over other firms (Zou et al., 2003). H2 posits that market-based innovations are positively associated with the export performance of SMEs in terms of economic export performance and strategic export performance. It is accepted as the β-estimate (0.532) from market-based innovations to export performance is significant with *p* < 0.05 (see Table 5; Figure 2). This finding supports the argument of Zhou et al. (2005) that market-based innovations provide a first-mover advantage to the firms, hence improving export performance. Market-based innovations depart from already available products and offer unique and simple products to target new markets. This orientation leads firms to enhance export performance. Furthermore, the impact of technology-based innovations on export performance is greater (β = 0.648) as compared to market-based innovation (β = 0.532).

**Table 5:** Influence of breakthrough innovations on export performance.

	Hypothesized paths	Estimate	SE	t-value	*p*-value	Results
H1	Technology-based innovations ➔ Export performance	0.282	0.041	6.852	***	Supported
H2	Market-based innovations ➔ Export performance	0.422	0.053	8.013	***	Supported

*** *p < 0.001.*

### Role of Institutional Environment

**Figure 3:**
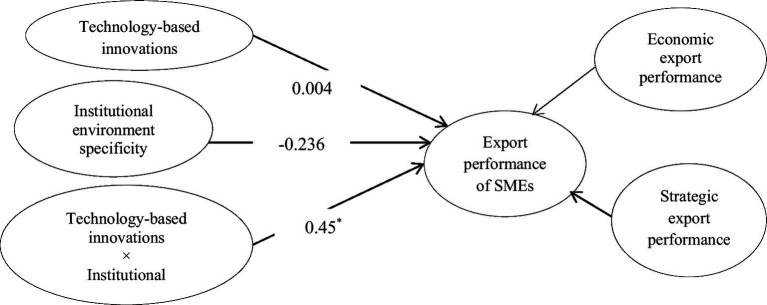
Moderating effect of institutional environment specificity on technology-based innovations–export performance link. ^*^p < 0.05; ^**^p < 0.01; ^***^p < 0.001.

**Figure 4:**
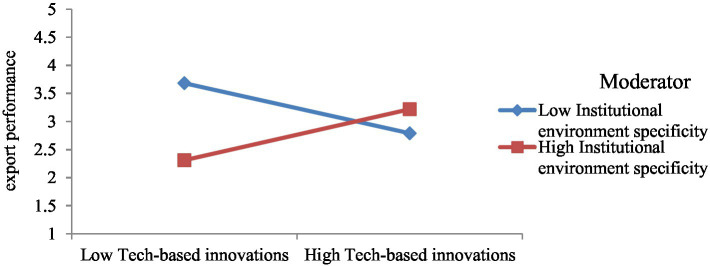
Interaction of model 1.

To test the moderating effect of institutional environment specificity and enforceability, we developed four separate structural models in AMOS. Model 1 includes interaction among technology-based innovations and institutional environment specificity (Figure 3). This model is used to test hypothesis 3a, which posits that institutional environment specificity improves the impact of technology-based innovations on the export performance of SMEs. It is accepted as the β-estimate (0.45) from interaction among technology-based innovations and institutional environment specificity to export performance is significant with *p* < 0.05. Besides, the interaction graph (Figure 4) shows that the more the institutional environment specificity, the greater the impact of technology-based innovations on export performance.

**Figure 5:**
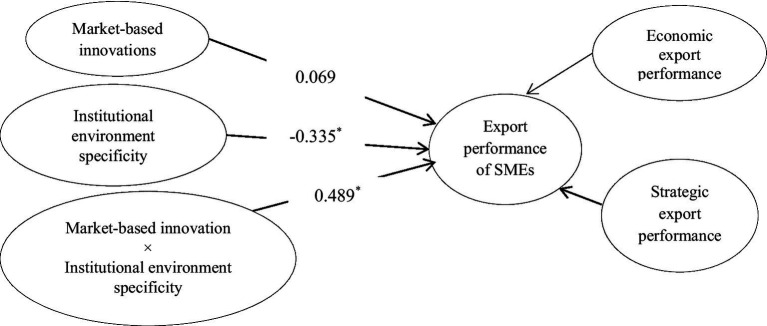
Moderating effect of institutional environment specificity on market-based innovation–export performance link. ^*^p < 0.05; ^**^p < 0.01; ^***^p < 0.001.

**Figure 6:**
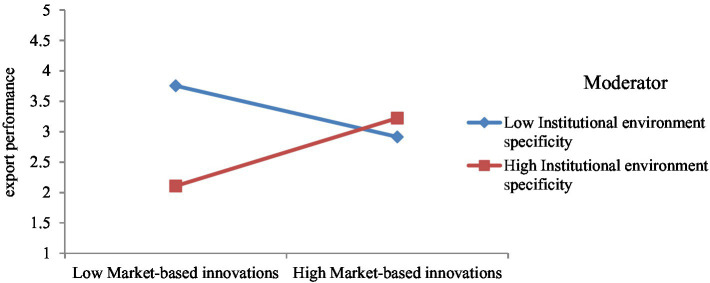
Interaction of model 2.

Model 2 includes interaction among market-based innovation and institutional environment specificity (Figure 5). This model is used to test hypothesis 3b, which posits that institutional environment specificity improves the impact of market-based innovation on the export performance of SMEs. It is accepted as the β-estimate (0.48) from interaction among market-based innovation and institutional environment specificity to export performance is significant with *p* < 0.05. Besides, the interaction graph (Figure 6) shows that the more the institutional environment specificity, the greater the impact of market-based innovation on export performance.

**Figure 7:**
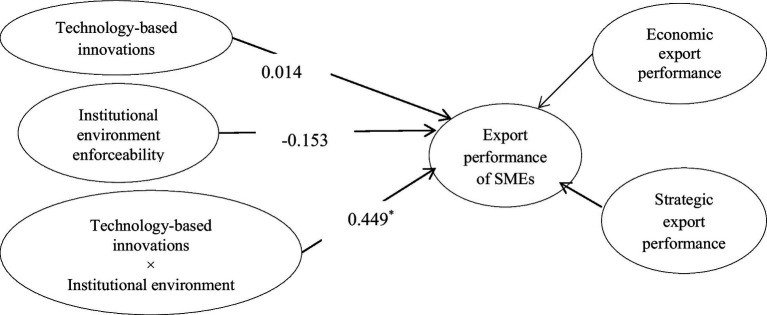
Moderating effect of institutional environment enforceability on technology-based innovations–export performance link. ^*^p < 0.05; ^**^p < 0.01; ^***^p < 0.001.

**Figure 8:**
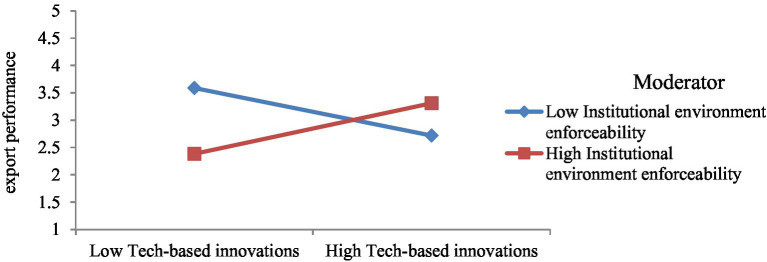
Interaction of model 3.

Model 3 includes interaction among technology-based innovations and institutional environment enforceability (Figure 7). This model is used to test hypothesis 4a, which posits that institutional environment enforceability improves the impact of technology-based innovations on the export performance of SMEs. It is accepted as the β-estimate (0.44) from interaction among technology-based innovations and institutional environment enforceability to export performance is significant with *p* < 0.05. Besides, the interaction graph (Figure 8) shows that more the institutional environment enforceability, the greater the impact of technology-based innovations on export performance.

**Figure 9:**
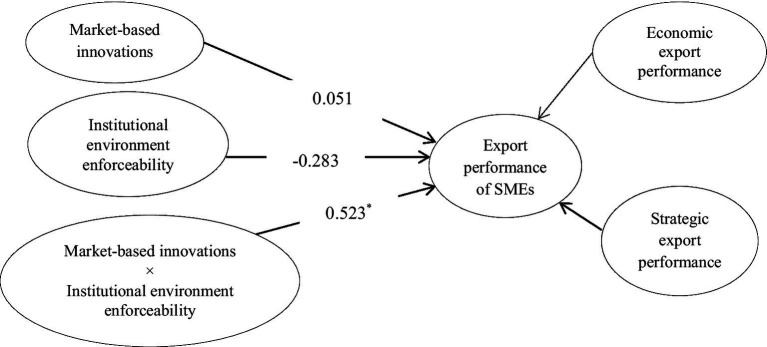
Moderating effect of institutional environment enforceability on market-based innovation–export performance link. ^*^p < 0.05; ^**^p < 0.01; ^***^p < 0.001.

**Figure 10:**
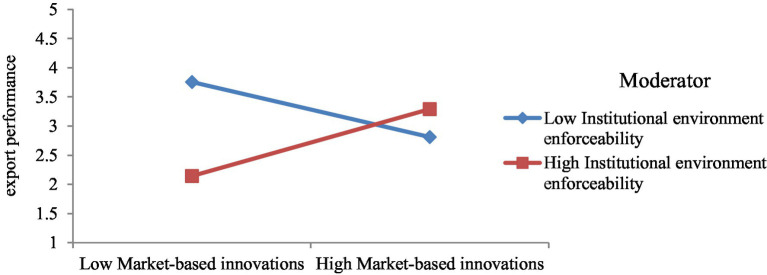
Interaction of model 4.

Model 4 includes interaction among market-based innovation and institutional environment enforceability (Figure 9). This model is used to test hypothesis 4b, which posits that institutional environment enforceability improves the impact of market-based innovations on the export performance of SMEs. It is accepted as the β-estimate (0.52) from interaction among market-based innovation and institutional environment enforceability to export performance is significant with p < 0.05. Besides, the interaction graph (Figure 10) shows that the more institutional environment enforceability, the greater the impact of market-based innovation on export performance.

## Discussion and Conclusion

### Theoretical Contributions

The current study aims to examine the influence of breakthrough innovation on the export performance of SMEs in a developing context. Also, the purpose of the current study is to assess the presence of the institutional environment (i.e., institutional environment specificity and enforceability), while examining the breakthrough innovations–export performance link. The findings reveal that technology-based innovations and market-based innovations enhance the export performance of SMEs, whereas technology-based innovations have a greater influence on export performance as compared to market-based innovations. Furthermore, we find that the institutional environment in terms of specificity and enforceability enables SMEs to enhance their export performance through breakthrough innovations.

The current study adds value to the export business literature, innovation, and institutional literature in the following three major ways. First, drawing on the institutional theory and institutional literature framework (Bruton et al., 2010; Hessels and Terjesen, 2010; Ngo et al., 2016; Amankwah-Amoah et al., 2018; Donbesuur et al., 2020), we extend the institutional environment by examining the moderating influence of institutional environment specificity and enforceability on the relationship between breakthrough innovations and export performance. We fulfill this gap by examining how institutional specificity and enforceability enhance the influence of technology-based innovations and market-based innovations on the export performance of developing SMEs. Also, the typology of breakthrough innovation comprises two distinctive innovation types, i.e., tech- and market-based innovation (Zhou et al., 2005). Therefore, the current study contributes significantly by offering a comprehensive moderating framework of the institutional environment in relation to breakthrough innovations–export performance link. Second, the current study contributes to the existing body of knowledge, which contends that innovation practices help firms in enhancing export performance (Azar and Ciabuschi, 2017; Silva et al., 2017; Tavassoli, 2018; Reçica et al., 2019; Edeh et al., 2020; Enjolras et al., 2020; Wu et al., 2021). Here, drawing on the dynamic capability theory, we demonstrate that breakthrough innovations in terms of tech- and market-based innovations are positively associated with the export performance of SMEs in a developing country. In developing countries, firms often face limited resources and thereby look for unique methods to use resources, leading to breakthrough innovations, while, in developed countries, where companies have better access to resources follow standard ways to use resources to comply with only regulations or business requirements. Finally, we tested our proposed model in the unique developing setting of Pakistan. In this regard, we extend the previous literature on the export performance of SMEs from developed economies (Silva et al., 2017) to developing contexts. This is an important contribution as little focus has been given to understand when and how SMEs in developing context enhance their export performance through their breakthrough innovations.

### Managerial Implications

This study has several implications for SME owners, policymakers, and those stakeholders who engage in export activities. First, our findings reveal that technology-based and market-based innovations improve the export performance of SMEs. Thus, SMEs can get maximum benefits from their export decisions by doing technology-based and market-based innovations. Specifically, SMEs can get better export performance by exploring their capabilities in developing technologically advanced products and unique products. It is often noted that SMEs in developing economies face difficulties and barriers in both local and international markets (El Makrini, 2017); however, their innovative capabilities can help them mitigate these barriers and participate in exports actively. Besides, SMEs can commit their resources coming through exports into R&D to bring more technology-based and market-based innovative products. Second, the institutional environment plays an important role in providing a safe path to innovative practices toward internationalization. In the developing context, we often observe that institutional voids and corruption hinder innovative activities. However, with globalization and advancement in technology, developing countries like Pakistan are working in maintaining a good institutional environment to protect innovations and inventions. Besides, managers appreciate institutional support as it can help them gain a competitive advantage in local and international markets. Therefore, institutional environment specificity and enforceability may induce confidence among the managers of SMEs, reduce market uncertainty, and provide freedom to operate in international markets. Given the economic benefits of the institutional environment, policymakers of the developing countries must specify and enforce a comprehensive legal framework for the protection of SMEs’ innovations and inventions.

### Limitations and Future Research Directions

The current study is limited in certain ways, setting avenues for future research. First, we have examined the moderating role of institutional environment specificity and enforceability among the relationship between breakthrough innovations and export performance. However, we did not include other attributes of the institutional environment such as institutional predictability and stability (Acemoglu and Johnson, 2005; Ngo et al., 2016). Therefore, future study is needed to consider these dimensions of the institutional environment for a more nuanced and comprehensive understanding of the impact of the institutional environment on breakthrough innovations–export performance link. Second, we consider export performance as a second-order construct, limiting us to determine the differential moderating effect of institutional environment on the link between breakthrough innovations, and strategic export performance, and economic export performance. Therefore, future studies can separately assess the moderating role of the institutional environment for strategic and economic export performance. Third, we have tested our model from a managerial perspective. Therefore, future study is required to examine and measure the institutional environment from other important stakeholders such as the government and policymakers. Fourth, as we collected data from only one developing country, it can be important to examine the study model in comparison with other developing and emerging countries. Finally, it can be interesting to evaluate the cultural and technological dimensions while examining the influence of breakthrough innovations on export performance.

## Data Availability Statement

The original contributions presented in the study are included in the article/Supplementary Material, further inquiries can be directed to the corresponding author.

## Ethics Statement

Ethical review and approval was not required for the study on human participants in accordance with the local legislation and institutional requirements. Written informed consent for participation was not required for this study in accordance with the national legislation and the institutional requirements.

## Author Contributions

The idea of the original draft belongs to HH and CV. The introduction literature review and empirical outcomes sections are written by HH and WO. HH, CV, WO, and MC helped in methodology, data analysis, writing, proofreading, and discussion. All authors read and approved the final manuscript.

## Conflict of Interest

The authors declare that the research was conducted in the absence of any commercial or financial relationships that could be construed as a potential conflict of interest.

## Publisher’s Note

All claims expressed in this article are solely those of the authors and do not necessarily represent those of their affiliated organizations, or those of the publisher, the editors and the reviewers. Any product that may be evaluated in this article, or claim that may be made by its manufacturer, is not guaranteed or endorsed by the publisher.

## Appendix A: Measures Detail

### Export Performance

#### Economic Export Performance (EXP)

Question: How do you evaluate your export venture results compared with your main competitors?(Scale: 1 = “much worse,” and 7 = “much better”).

**Table:**

EXP1	Export sales volume
EXP2	Profitability.
EXP3	Percentage of sales revenue derived from products introduced in this market during the past three years

#### Strategic Export Performance (EXP)

Question: When considering the selected export venture, what is your opinion concerning the following sentences?(Scale: 1 = “strongly disagree,” and 7 = “strongly agree”).

**Table:**

EXP1	This export venture has improved our global competitiveness.
EXP2	This export venture has strengthened our strategic position
EXP3	This export venture has significantly increased our global market share.

### Breakthrough Innovation

#### Technology-based innovations (Tech)

Question: When considering the product of the selected export venture, what is your opinion concerning the following sentences? (Scale: 1 = “strongly disagree,” and 7 = “strongly agree”).

**Table:**

Tech1	Our product is highly innovative, replacing an inferior alternative.
Tech2	Our product incorporates a radically new technological knowledge.
Tech3	High-quality technical innovations were introduced during the development of this product.
Tech4	Overall, our product is similar to our main competitors’ products
Tech5	The application of our product is totally different from that of our main competitors’ products.

#### Market-based innovation (Mar)

Question: When considering the product of the selected export venture, what is your opinion concerning the following sentences? (Scale: 1 = “strongly disagree,” and 7 = “strongly agree”).

**Table:**

Mar1	Our product concept is difficult for importers to evaluate or understand.
Mar2	The use of our product requires a major learning effort by importers.
Mar3	It takes a long time for importers to understand our product’s full benefits.
Mar4	Our product involves high switching costs for mainstream importers.

### Institutional Environment

#### Institutional Environment Specificity (IES)

Question: The respondents were directed to indicate to what extent they perceive about the existence of institutional rules and regulations about their innovations on a seven-point scale (1 for “non-existing” and 7 for “prevalent”).

**Table:**

IES1	Counterfeit products/services
IES2	Violation of intellectual property rights
IES3	Economic and commercial disputes between firms
IES4	Commercial fraud associated with new products/service introductions
IES5	Illegal cancelling of signed contracts

#### Institutional Environment Enforceability (IEE)

Question: Respondents to indicate to what extent they perceive about the level of enforcement regarding protection of their innovations on a seven-point scale (1 for “strongly disagree” and 7 for “strongly agree”).

**Table:**

IEE1	Counterfeit products/services
IEE2	Violation of intellectual property rights
IEE3	Commercial fraud associated with new products/service introductions
IEE4	Economic and commercial disputes between firms
IEE5	Monopoly in production and commercial activities
